# Binding Patterns of Rotavirus Genotypes P[4], P[6], and P[8] in China with Histo-Blood Group Antigens

**DOI:** 10.1371/journal.pone.0134584

**Published:** 2015-08-14

**Authors:** Xin Ma, Dan-di Li, Xiao-man Sun, Yan-qing Guo, Jing-yao Xiang, Wei-huan Wang, Li-xia Zhang, Qing-jiu Gu, Zhao-jun Duan

**Affiliations:** 1 China Railway Construction Corporation, Beijing Tiejian Hospital, Beijing, 100039, China; 2 National Institute for Viral Disease Control and Prevention, China CDC, Beijing, 102206, China; 3 Beijing Railway Center for Disease Control and Prevention, Beijing, 100038, China; University of Hong Kong, HONG KONG

## Abstract

Rotaviruses (RVs) are an important cause of severe gastroenteritis in children. It has been found that RV may recognize the histo-blood group antigens (HBGAs) as ligands or receptors and bind HBGAs in a type-dependent manner. In this study, we investigated the binding specificity of VP8* proteins from human rotaviruses (RV) that are prevalent in China including genotypes P[4], P[6], and P[8]. Through the saliva- and oligosaccharide-based binding assays, we found that the VP8* proteins of P[4] and P[8] RV showed similar reactivity with the Le^b^ and H type 1 antigens, while P[6] RV weakly bound the Le^b^ antigen. These findings may facilitate further research into RV host specificity and vaccine development.

## Introduction

Rotaviruses (RVs) belonging to the reovirus family are one of the most common causes of acute gastroenteritis in children. RVs are estimated to cause 453,000 deaths annually, mostly in low- and middle-income countries[1, 2].Currently, the WHO recommends two effective vaccines: Rotarix, which is an attenuated monovalent G1P[8] human RV strain reassortant vaccine, and Rotateq, which is a pentavalent (G1–4, P[8]) bovine-human reassortant vaccine[3, 4]. Since there is no effective treatment for rotavirus viral gastroenteritis, vaccination is of great importance for the RV prevention.

RV is non-enveloped and has an icosahedral structure consist of three concentric protein layers surrounding the core genome. The RV genome consists of eleven segments of double-stranded RNA encoding six structural proteins (VP1–4, VP6, and VP7) and six nonstructural proteins (NSP1–6) [5]. Two major structural proteins on the outmost layer, VP4 (protease-sensitive protein) and VP7 (glycoprotein), define RV P and G genotypes, respectively [6]. RV strains are genetically diverse and can be classified into 27 G and 35 P genotypes currently [7]. Epidemiological studies have found that four G genotypes (G1–G4) and three P genotypes (P[4], P[6], and P[8]) represent the major sources of human RV infection globally (>95% of human RV infections) [8].

VP4 can be cleaved through proteolysis into two subunits, VP5* and VP8* [9–11]. VP8*, the global head of the spike, is believed to participate in the attachment of the viruses to host cells [12, 13]. Viral infection mainly depends on the recognition of host cell receptors [14]. Some animal RV strains are sialic acid (SA)-dependent recognizing terminal SAs on the cell surface, while most animal and human RV strains are SA-independent [15, 16]. Many studies have been focused on the attachment factors for these SA-independent strains. Recently, it was reported that RVs could recognize histo-blood group antigens (HBGAs) as attachment factors or potential receptors, similar to human noroviruses (NoVs) [17–20].

HBGAs are complex carbohydrates which exist as free oligosaccharides in biologic fluids such as saliva, milk, and blood[21]. They are also present on red blood cells and epithelia of the genitourinary, respiratory, and digestive tracts[22, 23]. HBGAs are synthesized through the addition of monosaccharide to disaccharide precursors. This process is catalyzed by glycosyltransferases, which are encoded by the ABO, Lewis, and secretor gene families. The enzyme FUT2 is responsible for the formation of H antigen. Individuals who can produce ABH antigens are called secretors. Meanwhile, mutations in the *FUT2* gene that inactivate the enzyme result in the devoid of ABH antigens in biologic fluids or on the epithelial cell surfaces and these individuals are called non-secretors. *FUT3* gene is mainly involved in the synthesis of Lewis antigen, for example Lewis a (le^a^) or Lewis x (le^x^) in non-secretors and Lewis b (le^b^) or Lewis y (le^y^) in secretors.

VP8* proteins played an important role in the interaction with HBGAs. Moreover, this interaction was found to be genotype dependent and strain specific. It was reported that P[4] and P[8], two closely related P genotypes showed binding to the Le^b^ and H-type 1 antigens, while P[6] only bound to H-type 1[17]. Thus, the sialic acid-independent human RVs may recognize HBGAs as receptors. These findings were further confirmed by the crystallographic study of RV P[14] VP8* and A-HBGA[18]. The complex structure shows that A-HBGA interacts with the VP8* protein at the same location as the SA in the VP8* of animal RVs and suggests how subtle amino acid changes within the binding cavity allow for receptor switching. Furthermore, the research of the role of HBGAs in RV pathogenesis shows that P[14] rotavirus HAL1166 and the related P[9] human rotavirus K8 bind to A-type HBGAs and human rotaviruses DS-1 (P[4]) and RV-3 (P[6]) also use A-type HBGAs for infection, which indicate that human rotaviruses may use A-type HBGAs as receptors[24]. However, human P[8] rotavirus Wa does not recognize A-type HBGAs. These results show the complexity of the interactions between the RVs and HBGAs. Since only several RV strains were used to study the binding pattern and there are also variations between different RV strains, it remains uncertain that are all the strains in the same genotype shows similar HBGAs binding specificities.

In this study, we investigated the receptor binding specificity of prevalent RV strains isolated directly from the stool sample in China including P[4], P[6] and P[8] genotypes. The VP8* proteins of five different RV strains, 11221075 (P[8]), 11221345 (P[8]), 5311142 (P[6]), 11151099 (P[4]) and 11151328 (P[4]), were expressed and purified. For the saliva binding assay, 159 saliva samples were collected and the HBGA phenotypes were determined. The binding of VP8* proteins to synthetic oligosaccharides representing specific HBGAs were also examined. Our study provides the basis for the HBGAs binding research of RV strains in China, which is valuable for the vaccine development and vaccine application.

## Materials and Methods

### Ethics statement

The study was approved by the Medical Ethics Committee at National Institute for Viral Disease Control and Prevention, China CDC, Beijing, China. A written informed consent was obtained from the parents of 5 diarrhea children for stool samples. Written informed consents were obtained from 59 healthy undergraduate volunteers and the parents before enrollment of 100 primary school students for saliva samples in the study. The animal study proposal was approved by the Institutional Animal Care and Use Committee (IACUC) of National Institute for Viral Disease Control and Prevention, China CDC with the permit number: 20140219002. All rabbits experimental procedures were performed in accordance with the Regulations for the Administration of Affairs Concerning Experimental Animals approved by the State Council of People’s Republic of China.

### Sample selection and processing

The sequences of human RV strains of different P (VP4) genotypes were isolated directly from stool samples including 11221075 (P[8]), 11221345 (P[8]), 5311142 (P[6]), 11151099 (P[4]) and 11151328 (P[4]), (NCBI ID: KT162984-KT162988). The 10% stool suspension was prepared by mixing 0.1 g of stool and 0.9 ml of phosphate-buffered saline (PBS, pH 7.2) and centrifuging the tube at 8000 rpm for 5 min. The suspensions were saved at -20°C for later use.

### Viral RNA extraction and RT-PCR

Viral RNA was extracted from the stool suspensions using a viral RNA Mini Kit (Qiagen, Hilden, Germany). The primers (p541 and p556) were used as previously described [17]. The RV VP8* protein was amplified using a OneStep RT-PCR Kit (Qiagen).

### Gene cloning, protein expression, and purification

RV VP8* fragments (1–231 amino acids) were cloned into pGEX-4T-1 using the *Sal*I and *BamH*I endonuclease sites. The GST-VP8* fusion protein was expressed in *Escherichia coli* strain BL21 with 0.4 mM IPTG at 22°C. The fusion protein was purified using Glutathione Sepharose 4 Fast Flow (GE Healthcare Life Sciences, Little Chalfont, UK) according to the manufacturer’s protocol. The GST tag was cleaved with the thrombin enzyme at room temperature overnight and free VP8* protein was obtained.

### Western blot

The GST-fusion proteins were further confirmed by western blot. The five GST-VP8* proteins were transferred to PVDF membrane after SDS-PAGE electrophoresis. Then they were blocked with 8% nonfat milk and incubated for 1 h at room temperature. The mouse anti-GST monoclonal antibody was diluted at 1:1000. The goat anti-mouse IgG was added at 1:5000 as the secondary antibody. Then the GST-fusion proteins were showed by using HRP-DAB kit.

### Preparation of polyclonal serum

Six female SPF New Zealand white rabbits (20 weeks, about 2kg weight each) were purchased from Beijing B&M Biotech Company. They were immunized with six times with purified VP8* protein from RV strains 11151099, 11221075, and 5311142, every protein immunized two rabbits, respectively. Each rabbit was injected intradermally at multiple sites with 100 μg protein emulsified with Freund’s complete adjuvant in 1:1 ratio on the back of each rabbit. Two weeks after the initial injection, each animal was boosted five times by inoculation of 100 μg antigen mixed with an equal volume Freund’s incomplete adjuvant once a week. We detected the serum antibody titer by the indirect ELISA after the fifth immune procedure. As the titers were quantified, we proceeded with the sixth immune. One week after the final injection, The rabbits were injected 50mg/kg pentobarbital sodium via.IP. Then 120–160 ml blood was extracted from heart to obtain the serum. Rabbits were euthanized by exsanguination under anesthesia by pentobarbital. The antiserum was collected and stored at -80°C. Animal Experimental were reviewed and approved by ethics committee of National Institute for Viral Control and prevention, China CDC. All rabbits experimental procedures were performed in accordance with the Regulations for the Administration of Affairs Concerning Experimental Animals approved by the State Council of People’s Republic of China.

### Saliva collection and phenotyping

A total of 159 saliva samples were collected from 59 healthy undergraduate volunteers and 100 primary school students. The former were students at Beijing Union Medical College while the latter were students at Beijing Fengtai Elementary School. Saliva samples were collected by directing each individual to spit into a sterile 50 ml centrifuge tube. Then the samples were diluted, boiled, and stored at -20°C for HBGA phenotyping.

HBGA phenotyping of the saliva samples was done by enzyme immunoassays (EIAs) as previously described[21]. Briefly, saliva samples were diluted 1:1000 in PBS and coated on 96-well microtiter plates (Corning Inc., Corning, NY, USA). After blocking with 5% nonfat milk, monoclonal anti-Lewis a (Le^a^), anti-Lewis b (Le^b^), anti-Lewis x (Le^x^), anti-Lewis y (Le^y^), anti-A, and anti-B antigen antibodies were added. Corresponding secondary antibodies (HRP-conjugated goat anti-mouse IgM or IgG) were added after incubation for 1 h at 37°C. The color reaction was produced using a 3,3’,5,5’-tetramethylbenzidine (TMB) kit (Sigma-Aldrich, St. Louis, MO, USA). The optical density (OD) at a wavelength of 450 nm was read by an EIA spectrum reader (Thermo Fisher Scientific, Waltham, MA, USA).

### Binding of RV VP8* to carbohydrates in human saliva

Saliva-based binding assays were used to detect the binding of RV VP8* to carbohydrates in human saliva as previously reported [25]. We used 159 saliva samples for which the HBGA phenotype had been identified. The samples were boiled at 100°C for 10 minutes and diluted 1:1000 in PBS. The diluted samples were coated onto 96-well microtiter plates (Corning Inc.) overnight at 4°C. After blocking with 5% nonfat milk, free VP8* protein was added and incubated for 1 h at 37°C. The bound VP8* proteins were detected using rabbit serum anti-P[4],-P[6], and-P[8] antibodies diluted at 1:1000. HRP-conjugated goat anti-rabbit IgG (Abcam, Cambridge, UK) was added at 1:3000 as the secondary antibody. The signal intensities were displayed using a TMB kit and the OD at 450 nm was read as described above.

### Binding of RV VP8* to synthetic oligosaccharides

To test for oligosaccharide binding using EIAs, microtiter plates were coated with free VP8* at 10 μg/ml overnight at 4°C. After blocking with 5% nonfat milk, oligosaccharide-6-aminohexanoate (LC)-biotin or oligosaccharide-polyacrylamide (PAA)-biotin was diluted 1:500 and added to the microtiter plate at 4°C overnight. The bound oligosaccharides were detected by using HRP-conjugated streptavidin. Finally, the OD at 450 nm was read as described previously.

## Results

### Distribution of ABO, Lewis, and secretor types among 159 saliva samples

In order to identify the correlation between HBGAs and VP8*, the HBGA phenotypes of the saliva samples were determined by EIAs. Among the 159 volunteers, 46 were type A, 33 were type B, 12 were type AB, and 68 were type O. Meantime, it is reported that Le^b^ and/or Le^y^ positive individuals account for the most in the population [26]. In our experiment, 159 individuals were tested, 144 (91%) were secretors (Le^b+^ or Le^y+^) and 15 (9%) were non-secretors (Le^a+b-^ or Le^x+y-^).

### The VP8* proteins of RV strains P[4] and P[8] bind to Le^b^ and/or H1

**Fig 1:**
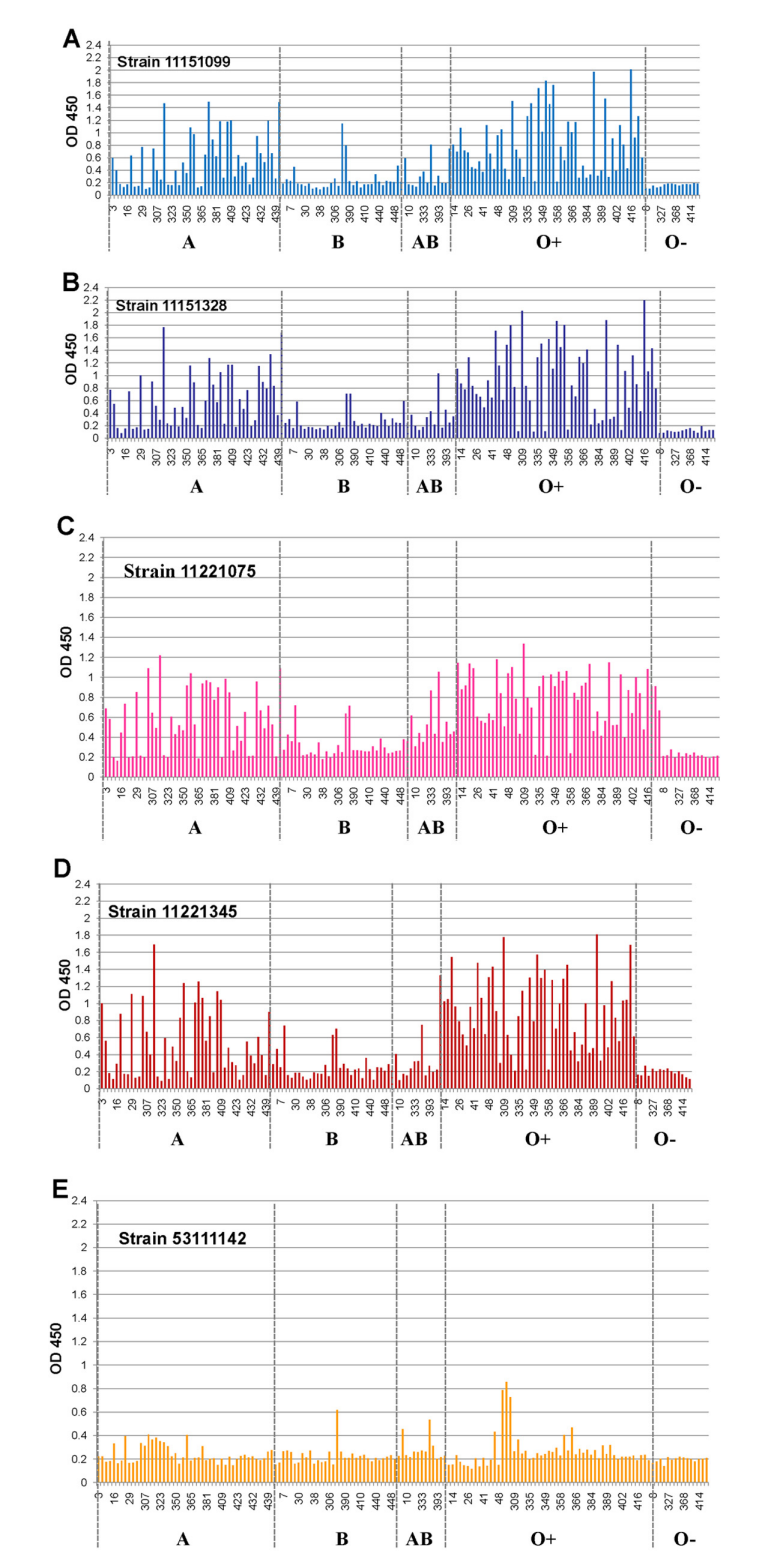
Binding assay of five RVs with HBGAs from the saliva of 159 individuals with various blood types. **P[4], P[6], and P[8] RVs were observed to bind to saliva from type A/B, AB, and O secretors (Le** ^**b**^ **positive).** Low-level binding of P[6] RV to saliva was also tested. A, 11151099 P[4] VP8*; B, 11151328 P[4] VP8*; C, 11221075 P[8] VP8*; D, 11221345 P[8] VP8*; E, 5311142 P[6] VP8*.

**Fig 2:**
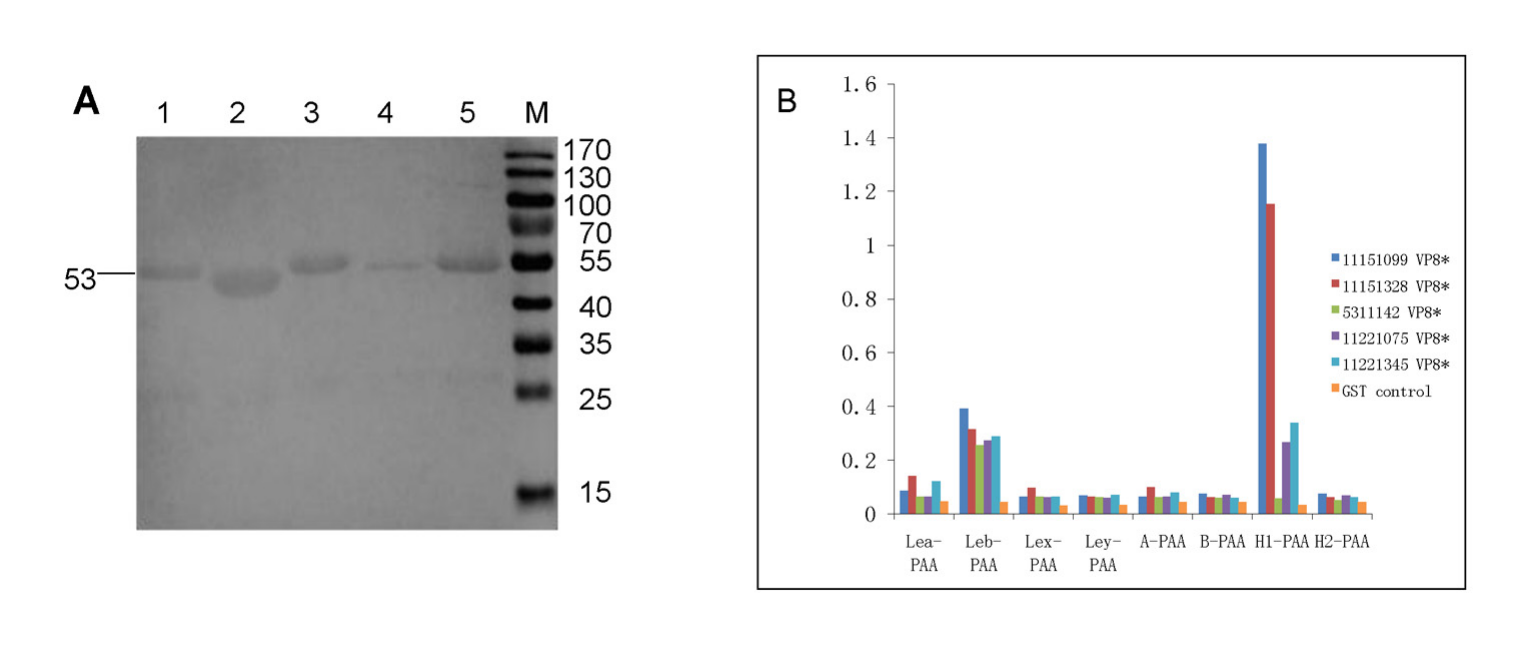
A, Western blot analysis of GST-VP8* recombinant proteins. 1, GST-11151099 P[4] VP8* recombinant protein; 2, GST-11151328 P[4] VP8* recombinant protein; 3, GST-5311142 P[6] VP8*recombinant protein; 4, GST-11221075 P[8] VP8* recombinant protein; 5, GST-11221345 P[8] VP8* recombinant protein; M, protein ladder marker. **B, Binding assay of five RVs with HBGAs of synthetic oligosaccharides.** P[4] and P[8] RVs were observed to specifically bind to Le^b^ and H1. Weak signals were detected for P[6] RV binding to Le^b^.

**Fig 3:**
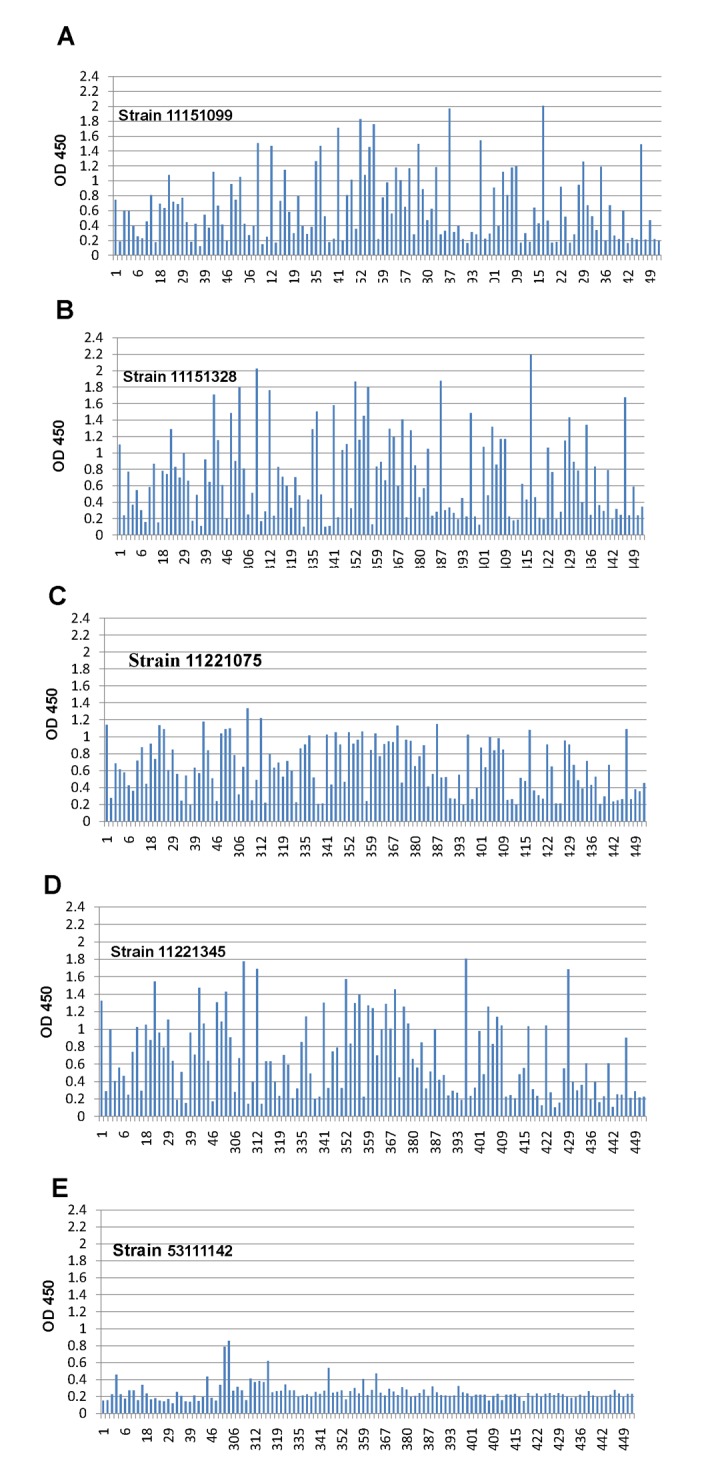
Binding assay of VP8* proteins from five RV strains to a panel of Le^b^-positive saliva samples from 159 individuals. P[4] and P[8] RVs clearly bound to Le^b^. Very weak signals were detected for P[6] RV binding to Le^b^.

In this study, we used saliva- and oligosaccharide-based binding assays to examine the binding of RV VP8* proteins to HBGAs. When the highly purified VP8* proteins of RV strains P[4] and P[8] were examined, clear binding to type A/B/AB and type O saliva samples was shown (Fig 1A, 1B, 1C and 1D). The VP8* proteins were obtained successfully as shown by the SDS-PAGE (Fig 2A). The interaction was further clarified by binding to Le^b^-positive saliva (Fig 3A, 3B, 3C and 3D). In the oligosaccharide binding assay, binding to H type 1 (H1) and Le^b^ antigens was clearly observed (Fig 2B). The oligosaccharide binding results not only confirmed the saliva binding assays, but also showed that the VP8* proteins of P[4] and P[8] could bind to H1.

### The VP8* protein of RV P[6] bind to the Le^b^

For the VP8* protein of RV P[6], no obvious binding to type A/B/AB and type O saliva samples was shown in the saliva-based binding assays. However, the signal of binding to Le^b^ antigens in the saliva samples was weakly detected (Figs 1E and 3E). Furthermore, in the oligosaccharide binding assay, weak binding was also observed between VP8* and Le^b^ (Fig 2B), which indicates the P[6] VP8* protein may bind to the Le^b^.

### The VP8* proteins of RV P[4], P[6], and P[8] do not recognize Le^a^ and/or Le^x^

In the analysis of the human saliva samples, there was no obvious binding between the free VP8* proteins to O^**-**^ saliva which contains Le^a^ and/or Le^x^ antigens (Fig 1). Moreover, in the oligosaccharide-based binding assays, no binding to Le^a^ and Le^x^ was detected (Fig 2B). These indicate that the three P genotype RVs may not be capable of infecting non-secretors.

## Discussion

In this study, we isolated five human RV strains belonging to genotypes P[4], P[6], and P[8], which are prevalent in China, and successfully obtained the VP8* proteins. Besides, we elicited an association between these RV VP8* proteins and HBGAs. In previous studies, RVs have been found to recognize diverse human HBGAs and show different HBGAs binding patterns. Here, we further confirm that RVs may recognize HBGAs in a genotype-dependent manner.

The finding that the VP8* proteins of RV P[4] and P[8], which are prevalent in China, can recognize Le^b^ and/or H1 is consistent with the previous research [17]. The VP8* sequence alignment of the P[4] RV strains (11151099 and 11151328) with DS-1 (P[4]) and the P[8] (11221075 and 11221345) with Wa (P[8]) shows that the inter-genotype sequence identity is about 97%. Furthermore, the key amino acids at position 101, 146, 155, 187, 188, 189, 190, 191, which are involved in the sialic acid-P[3] or A-HBGA-P[14] binding in the previously reported crystal structures are the same among all these RV strains except the amino acid at position 189 (varied between Ser and Asn) [18,27], indicating that these VP8* proteins may share similar binding modes as illustrated in the functional assay. Secretor antigens are present in about 80% of the population in Europe and North America [28, 29]. In many countries, more than 90% of cases of diarrhea following RV infection are caused by P[4] and P[8] RVs [30]. It was also observed that P[8] RVs infect only secretor-positive children, as demonstrated by epidemiological studies from France, Vietnam, and Burkina Faso [22,31,32]. Therefore, it is presumed that an individual’s secretor status may be associated with human RV infection.

In the Le^b^-positive saliva binding assay of P[6] RV VP8*, weak binding was shown. Furthermore, in the oligosaccharide-based binding assays, signals of binding to Le^b^ antigens were detectable though very weak. It indicates that the VP8* protein of P[6] RV may recognize Le^b^. The binding pattern of P[6] in this study is somewhat different from that of American RV strains, which binds to H1 [17]. However, data from recent epidemiological studies from Vietnam and Burkina Faso showed low-level Le^b^ expression in P[6]-infected children and a 33% infection rate by P[6] RV in Lewis-positive children [22, 32]. The different interaction patterns for P[6] RV and HBGAs may result from the diversity of RV strains in Africa and Asia compared with those in the USA and the sequence identity of VP8* protein between P[6] in our lab and ST3 P[6] is about 92%. However, in view of the binding of P[6] RV and HBGAs in the study was quiet low and only free VP8* protein was employed, further research is still needed. Moreover, it is necessary to obtain additional P[6] RV-positive samples for study in order to establish the correlation between P[6] RV infection and HBGA types. Recently, it was indicated that A-type HBGAs may be receptors for human RVs by the RV pathogenesis assay in the virus binding level which found that P[14] RV HAL1166, P[9] human RV K8, human RV DS-1 (P[4]) and RV-3 (P[6]) all bind to A-HBGAs. In our work, we investigate the interaction between RV and HBGAs in the protein level. It still needs much more work to clarify the complex interactions both in vivo and in vitro.

In addition, the correlation between the three major P genotypes and non-secretor antigens through VP8* proteins can also be elicited in this study. There was no obvious binding of P[4], P[6], and P[8] RVs to O^**-**^ saliva in the saliva-based binding assays and also no signal to Le^a^ or Le^x^ in the oligosaccharide binding assay, suggesting that RVs of these three P genotypes may not infect non-secretors.

In conclusion, this is the first study to illustrate the binding patterns of epidemic RV strains in China with human HBGAs. The interactions between VP8* proteins and HBGAs will enhance the understanding of the correlation between RVs and host cells, which are also essential for RV vaccine development.
